# Incidence and risk factors of antiretroviral treatment failure in treatment-naïve HIV-infected patients at Chiang Mai University Hospital, Thailand

**DOI:** 10.1186/1742-6405-8-42

**Published:** 2011-11-07

**Authors:** Nitta Khienprasit, Romanee Chaiwarith, Thira Sirisanthana, Khuanchai Supparatpinyo

**Affiliations:** 1 Department of Medicine, Faculty of Medicine, Chiang Mai University, Chiang Mai, Thailand; 2 Research Institute for Health Sciences, Chiang Mai University, Chiang Mai, Thailand

**Keywords:** Incidence, Risk factors, Antiretroviral Treatment Failure, Treatment-naïve

## Abstract

**Background:**

The use of combination antiretroviral therapy (cART) has become a standard of care for the treatment of HIV infection. However, cost and resistance to cART are major obstacles for access to treatment especially in resource-limited settings. In this study, we aimed to determine the incidence and risk factors of treatment failure in a cohort of treatment-naïve Thai HIV-infected patients.

**Methods:**

A retrospective cohort study was conducted among HIV-infected patients initiating their first cART at Chiang Mai University Hospital, Thailand.

**Results:**

From January 2002 to December 2008, 788 patients were enrolled; 365 were male (46.3%), and the mean age was 37.9 ± 8.6 years. The median baseline CD4 count was 57.7 cells/mm^3 ^(IQR 22, 127). GPO-VIR^® ^(a fixed-dose combination of lamivudine, stavudine, and nevirapine) was the most common prescribed cART (657 patients, 83.4%). Seventy-six patients developed virological failure given the cumulative incidence of 9.6%. The incidence of virological failure was 2.79 (95% CI 2.47, 3.14) cases per 100 person years. Poor adherence was the strongest predictor for virological failure. Of 535 immunologically evaluable patients, 179 (33.5%) patients developed immunological failure. A low CD4 cell count at baseline (< 100 cells/mm^3^) and the increment of CD4 cell count of < 50 cell/mm^3 ^after 6 months of cART were the predictors for immunological failure (p < 0.001).

**Conclusions:**

This study demonstrated that even in resource-limited settings, the high rate of success could be expected in the cohort with good and sustainable drug adherence. Poor adherence, older age, and low baseline CD4 cell count are the predictors for unfavorable outcome of cART.

## Introduction

By the year 2010, the Joint United Nations Program on HIV/AIDS (UNAIDS) estimate that there are 1,138,020 people living with HIV/AIDS in Thailand [1]. The mathematic model describing the epidemic trends using the Asia Epidemic Model software projected that there will be 10,835 new HIV cases each year [1]. In Thailand, it was only after the establishment of the National Access to Antiretroviral Program for People living with HIV/AIDS (NAPHA) in 2002 that combination antiretroviral therapy (cART) became widely available free of charge throughout the country [2]. In a previous study from Thailand, treatment with GPO-VIR^® ^(a locally-produced generic fixed-dose combination of stavudine, lamivudine, and nevirapine) resulted in 62.7% and 93.3% of 90 HIV treatment-naïve patients achieving undetectable HIV viral load at 24 and 48 weeks, respectively [3]. Despite the significant reduction in morbidity and mortality among HIV-infected patients receiving cART, a considerable number of patients fail to achieve a sustained virological and immunological response to therapy [4-7]. The incidence and predictors of treatment failure have generally been analyzed worldwide, [8-10] however, these results may not be applicable to more heterogeneous cohorts such as in Thailand, where non-nucleoside reverse transcriptase inhibitor (NNRTI)-based regimen is the first line of treatment, the majority of HIV-1 virus is subtype E (CRF01_AE), and cART is usually initiated at a very low CD4 count. The data regarding treatment failure from Thailand are still limited. Furthermore, the number of long-term cART treatment cohorts is scarce. Hence, our primary objective was to study the incidence and risk factors for treatment failure in a large cohort in northern Thailand. Such data would assist physicians in Thailand as well as in similar resource limited settings predicting and detecting treatment failure earlier in the course of cART.

## Materials and methods

### Study design and population

A retrospective cohort study was conducted at Chiang Mai University Hospital, an 1800-bed, tertiary-care hospital in Northern Thailand. All patients with documented HIV infection who were under medical care at the HIV Clinic and met the following criteria were included: 1) ≥ 15 years of age, 2) naïve to cART, 3) initiated their first cART regimen between January 1, 2002 and December 31, 2008, and 4) had at least 2 consecutive visits after cART initiation.

### Data collection

Baseline demographic data at the time of cART initiation were recorded including sex, age at cART initiation, race (Thai or hill-tribe), residential area (urban or rural area), type of health insurance (government fund, social security fund, Global Fund for AIDS, TB, and malaria, no funding support), and route of HIV transmission (men who have sex with men (MSM), heterosexual, injection drug user (IDU)). Medical data included diagnosis of AIDS-defining illness prior to cART initiation, cART regimen, date when cART was started, body weight, and self-reported adherence to cART was evaluated by asking patients the number of missing doses of antiretroviral drugs between visits. Baseline laboratory data included CD4 cell count, hemoglobin level, hepatitis B surface antigen (HBs Ag), anti-hepatitis C virus antibody (anti-HCV) and plasma HIV-1 RNA level (if available). Patients were followed up every 2-4 weeks during the first few months of cART initiation, and every 3-6 months thereafter. CBC and CD4 cell counts were measured every 6 months whereas plasma HIV-1 RNA quantification was done every 6 to 12 months.

### Definitions

cART was defined as a treatment regimen containing a non-nucleoside reverse transcriptase inhibitor (NNRTI) (either nevirapine or efavirenz), or a protease inhibitor (PI) plus 2 nucleoside reverse transcriptase inhibitors (NRTIs), or triple NRTIs (zidovudine/lamivudine/abacavir).

Adherence rate (%) was defined as (total doses taken/total doses prescribed) × 100, and was classified as 100%, 95-99%, 90-94%, 80-90%, and < 80% categories. Due to missing data on adherence rate at the beginning of our cohort, it was available in the majority of patients only in the last 4 years of follow-up.

Treatment failure was defined as either virological failure or immunological failure. Virological failure was defined as 1) incomplete virological response, i.e., confirmed HIV-1 RNA > 50 copies/ml after 48 weeks of cART, or 2) virological rebound, i.e., confirmed HIV-1 RNA > 50 copies/ml after initial virological suppression. Immunological failure was defined as failure to achieve CD4 cell count > 350 cells/mm^3 ^despite virological suppression (HIV-1 RNA < 50 copies/ml) after ≥ 2 years of cART.

### Statistical analysis

The cumulative incidence of treatment failure was calculated by the number of patients who had treatment failure over the number of patients who received cART during the study period. The incidence rate of virological failure was calculated by the number of patients who developed virological failure over the person-time from cART initiation to the development of virological failure. Patients who did not develop virological failure were censored as of December 31, 2008.

Baseline characteristics between groups of patients with and without treatment failure were compared using Student's t test for normally distributed continuous data, or Mann-Whitney U tests for non-normally distributed continuous data. Chi-square or Fisher exact tests were used to compare categorical data as appropriate.

Logistic regression models were used to identify covariates that were associated with treatment failure. Factors that were significant at the p-value < 0.10 in the univariate analysis were then evaluated in multivariable model using forward stepwise procedures.

All analyses were performed using Stata statistical software: Release 10.0 Stata Corporation, College station, TX, 2007). All p-values were two-sided. A level of significance of 0.05 was used to guide interpretation of relationships in final multivariable model.

The study was approved by the Faculty of Medicine, Chiang Mai University Ethical Committee.

## Results

### Patient Characteristics

**Figure 1:**
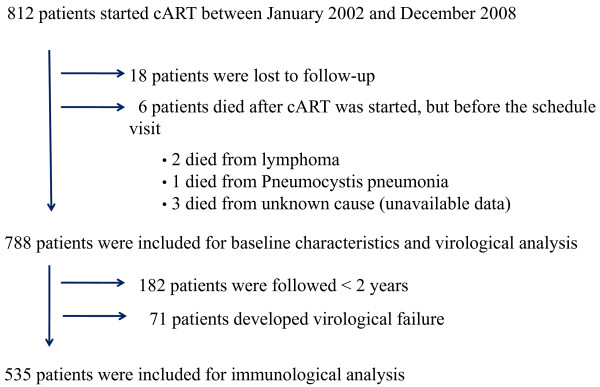
**Diagram of HIV-infected patients enrolled at HIV Clinic of Chiang Mai University Hospital from January 2002-December 2008**.

During the study period from January 2002 to December 2008, 812 treatment-naive HIV-infected patients were treated with cART and followed up at the HIV Clinic of Chiang Mai University Hospital. 24 patients (3%) were excluded from the analysis; 6 of these died shortly after cART initiation from lymphoma (2 patients), *Pneumocystis *pneumonia (1) and unknown causes (3). 18 patients were lost to follow-up after a median time of 18 weeks after cART initiation. The remaining 788 patients (97.0%) were followed up regularly for at least one year and fulfilled the inclusion criteria for the analysis of virological failure. Among 606 patients who were followed up for at least 2 years, 71 patients developed virological failure; therefore, there were 535 patients included in the analysis of immunological failure (Figure 1). Table 1 shows the baseline characteristics of the 788 patients in this cohort. NNRTI-based regimen was initiated in 774 patients (98.2%) and GPO-VIR^® ^was the most commonly prescribed first line regimen (84.1%).

**Table 1:** Baseline characteristics of 788 HIV-infected patients enrolled at HIV Clinic of Chiang Mai University Hospital from January 2002 to December 2008

Characteristics	Values
Male	365 (46.3)
Age (years)	39 ± 8.6
Route of transmission (N = 775)	
Heterosexual	740 (95.5)
Homosexual	27 (3.5)
IVDU	5 (0.6)
Unknown	3 (0.4)
Race (Thai)	764 (97.0)
Baseline body weight (kilograms) (N = 628)	54.4 ± 10.4
Residential area (Urban area)	597 (82.3)
Health insurance (N = 777)	
No	46 (5.9)
Government fund	142 (18.3)
Social security fund	418 (53.8)
Global Fund for AIDS, TB, Malaria	171 (22.0)
Combination antiretroviral therapy (cART)	
Nevirapine-based	657 (83.4)
Efavirenz-based	117 (14.8)
Protease inhibitor-based	8 (1.0)
Zidovudine/lamivudine/abacavir	6 (0.8)
Opportunistic infections (OIs) (N = 782)	490 (62.7)
OIs before cART	434 (55.5)
Tuberculosis	198 (25.3)
Adherence ≥ 95%	754 (95.7)
Laboratory findings	
Baseline CD4 cell counts (median, IQR) (cells/mm^3^)	57.7 (22, 127)
Baseline CD4 cell counts < 100 cells/mm^3^	525 (66.6)
Baseline CD4 cell counts 100-199 cells/mm^3^	195 (24.7)
Baseline CD4 cell counts ≥ 200 cells/mm^3^	68 (8.7)
Baseline Hemoglobin level (mg/dL)	11.2 ± 1.9
Positive anti-HCV antibody (N = 599)	61(10.2)
Positive HBs-antigen (N = 608)	66 (10.9)

Values are number (%) or mean ± standard deviation, unless otherwise specified

### Outcome of cART

During the study period of 6 years and a median follow-up duration of 3.63 years (IQR 1.66, 5.19), 76 of 788 patients developed virological failure giving the cumulative incidence of 9.6% by on-treatment analysis. The cumulative incidence of virological failure was 12.3% (100 of 812 patients) by intention-to-treat analysis. The incidence rate of virological failure was 2.79 cases (95% CI 2.47, 3.14) per 100 person-years. In 712 patients who had no virological failure, the median CD4 cell count increased from the baseline of 60 (IQR 24, 130) cells/mm^3 ^to 222 (IQR 150, 310) cells/mm^3 ^in 12 month (p < 0.001). In 76 patients with virological failure, the median CD4cell count increased from the baseline of 42 (IQR 20, 100) cells/mm^3 ^to 187 (IQR 120, 210) cells/mm^3 ^(p < 0.001).

Among 535 patients who were able to follow up for at least 2 years and included in the analysis of immunological failure, 179 patients (33.5%) experienced immunological failure despite virological suppression. After 2 years of cART initiation, the increment of median CD4 cell count from baseline was 152 (IQR 105, 214) cells/mm^3 ^and 294 (IQR 209, 370) cells/mm^3 ^among those with and without immunological failure, respectively (p < 0.001). The proportion of patients achieving CD4 cell count of ≥ 350 cell/mm^3 ^after 2 years of cART was 200/339 patient (59.0%) and 154/193 patients (79.8%) among those initiated cART at CD4 cell count of < 100 cell/mm^3 ^and ≥ 100 cells/mm^3^, respectively (p < 0.001).

### Risk factors for virological failure

Besides age at the cART initiation (36.6 ± 7.9 years among those with virological failure and 39.3 ± 8.6 years among those without virological failure, p = 0.011), there was no difference in baseline characteristics between 2 groups including sex, race, route of HIV transmission, residential area, type of health insurance, OIs before cART, cART regimen, and body weight. In addition, baseline laboratory data were not different between groups including CD4 cell count at cART initiation.

The univariate and multivariate analyses to determine the predicting factors for virological failure were shown in Table 2. In multivariate analysis, factors associated with virological failure were adherence of < 95% [OR 86.49 (31.19-239.80), p < 0.001] and age < 40 years [OR 2.28 (1.22-4.25), p = 0.04].

**Table 2:** Comparisons of clinical parameters to determine risk factors of virological failure among 788 HIV-infected patients

Variables	Univariate analysis		Multivariate analysis	
	
	Odds Ratio (95% CI)	P-value	Odds Ratio (95%CI)	P-value
Male vs. female	0.95 (0.65-1.69)	0.847	-	-
Age < 40 vs. ≥ 40 years	2.06 (1.23-3.46)	0.006	2.28 (1.22-4.25)	0.040
Heterosexual vs. non-heterosexual transmission	0.49 (0.20-1.23)	0.125		
Urban vs. rural area	1.18 (0.66-2.10)	0.581	-	-
No health insurance vs. had health insurance	0.77 (0.23-2.57)	0.671		
OIs before cART vs. no OIs before cART	0.84 (0.53-1.36)	0.488	-	-
Baseline CD4 cell count < 100 vs. ≥ 100 cells/mm^3^	1.56 (0.91-2.69)	0.106	-	-
Positive HCV antibody vs. negative HCV antibody	0.93 (0.35-2.44)	0.887	-	-
Positive HBs Ag vs. negative HBs antigen	1.25 (0.54-2.89)	0.603	-	-
Adherence < 95% vs. ≥ 95%	87.24 (32.29-235.72)	< 0.0001	86.49 (31.19-239.80)	< 0.001

Abbreviations: OI, opportunistic infection; cART, combination antiretroviral therapy; HCV, hepatitis C virus; HBs Ag, hepatitis B surface antigen

### Risk factors for immunological failure

Among those with and without immunological failure, 92 of 179 (51.4%) vs. 138 of 356 (38.8%) were male (p = 0.005) and 111 of 179 (62.0%) vs. 186 of 356 (52.3%) had OIs before initiation of cART (p = 0.032). There was no difference in other baseline characteristics between 2 groups including age, race, route of HIV transmission, residential area, type of health insurance, cART regimen, and body weight. Baseline laboratory data were also not different between groups, except for CD4 cell count at cART initiation and HCV serology. Among those with and without immunological failure, the median CD4 cell count was 44 (IQR 20, 87) cells/mm^3 ^vs. 78 (33, 150) cells/mm^3 ^(p < 0.001) and 23/138 (16.7%) vs. 28/283 (9.9%) had positive HCV antibody (p = 0.046).

Table 3 shows the univariate and multivariate analyses to determine the predicting factors for immunological failure. In univariate analysis, factors associated with immunological failure were male, history of OIs prior to cART initiation, lower baseline CD4 cell counts, and lower CD4 cell increase after 6 months of cART. The baseline CD4 cell count of < 100 cells/mm^3 ^[OR 2.69 (1.73-4.18), p < 0.001], and CD4 cell increase of < 50 cell/mm^3 ^after 6 months of cART [OR 2.39 (1.54-3.73), p < 0.001], remained significant in the multivariate analysis.

**Table 3:** Comparisons of clinical parameters to determine risk factors of immunological failure among 535 HIV-infected patients

Variables	Univariate analysis		Multivariate analysis	
	
	Odds Ratio (95% CI)	P-value	Odds Ratio (95% CI)	P-value
Male vs. female	1.67 (1.16-2.40)	0.006		
Age < 40 vs. ≥ 40 years	0.92 (0.65-1.32)	0.667	-	-
Heterosexual vs. non-heterosexual transmission	5.14 (0.65-40.50)	0.120	-	-
Urban vs. rural area	0.86 (0.57-1.31)	0.492	-	-
No health insurance vs. had health insurance	0.73 (0.23-2.33)	0.597	-	-
OIs before cART vs. no OIs before cART	1.49 (1.03-2.15)	0.032	-	-
Baseline CD4 cell count < 100 vs. ≥ 100 cells/mm ^3^	2.74 (1.81-4.15)	< 0.001	2.69 (1.73-4.18)	< 0.001
CD4 cell increase of < 50 vs. ≥ 50 cells/mm^3 ^after 6 months of cART	2.21 (1.44-3.39)	< 0.001	2.39 (1.54-3.73)	< 0.001
Positive HCV antibody vs. negative HCV antibody	1.823 (1.015-3.30)	0.048	-	-
Positive HBs Ag vs. negative HBs Ag	1.30 (0.67-2.51)	0.434	-	-
Adherence rate < 95% vs. ≥ 95%	2.87 (0.26-32.1)	0.392	-	-

Abbreviations: OI, opportunistic infection; cART, combination antiretroviral therapy; HCV, hepatitis C virus; HBs Ag, hepatitis B surface antigen

## Discussion

This is a large cohort of HIV-infected patients with middle or low socioeconomic status from a tertiary care hospital in northern part of Thailand. During the study period of 6 years and a median follow-up duration of 3.63 years (IQR1.66, 5.19), the cumulative incidence of treatment failure was 12.3% and 9.6% by intention-to-treat and on treatment analyses, respectively. This result is similar to failure rates found in a previous study by Anekthanonon et al from Bangkok, Thailand (2.4% by on treatment and 19.8% by intention- to -treat analysis)[11] and in the same range of 3-27% from many cohorts [10,12,13]. The wide range of virological failure from many cohorts could be attributable to differences in definition of treatment and virological failure between studies as well as the length of follow-up. However, in this study in which majority of patients were infected with HIV-1 subtype CRF01_AE, the immunological and virological outcomes are comparable to those from many US and European cohorts in which majority of patients were infected with HIV-1 subtype B, and those from African cohorts in which majority of patients were infected with HIV-1 subtype C [10,14-16]. The incidence rate of virological failure in our study was 2.79 cases per 100 person-years which is relatively low compared to the range of 3.36 cases per 100 person-year to 13.6 cases per 100 person-years in other cohorts [9,17]. This high response rate may be from the very high adherence rate in our cohort.

We found that adherence of < 95% and age < 40 years were the independent predicting factors for virological failure. Although adherence has been demonstrated to be important in many clinical cohorts, [18-21]there is discordance of results depending on types of adherence measurement. In our study, the data on adherence were collected according to the patient's self report of the amount of medication taken between visits without standardized set of questions. Therefore, the result may not be completely reliable. However, it is still demonstrated to be an independent factor for treatment failure. The finding that younger age at cART initiation was the risk factor for virological failure is similar to other studies [14,22]. Increased age may be a marker for greater maturity, lifestyle stability, and disease-specific education; these factors are likely to affect long-term adherence to therapy [14,23]. However, younger age and poor adherence were both independent risk factors for virological failure.

The SWISS HIV Cohort Study showed that only 39% of individuals treated with cART reached CD4 T-lymphocyte count of 500 cells/mm^3 ^[15]. Many HIV cohorts demonstrated factors potentially affect the recovery of CD4 T- lymphocyte including age, HCV serology, duration of HIV-1 infection, CDC stage, baseline HIV-1 RNA levels, baseline CD4 count, and adherence of cART [8-10,13,24]. Our study found that there was significant lower proportion of patients achieving CD4 cell count of ≥ 350 cell/mm^3 ^after 2 years of cART between those with baseline CD4 cell count ≤ 100 cell/mm^3 ^and those with higher baseline CD4 count (59.0% vs. 79.8%). It has been hypothesized that patients who initiated cART late in the course of disease may have thymic damage and limitation of T cell production and proliferation [25,26]. We could also demonstrate that the increment of the CD4 cell count of < 50 cells/mm^3 ^after the first 6 month of cART may predict immunological failure. Failure to achieve early CD4 recovery could be another signal of thymic damage. Our study did not find the difference in CD4 recovery between sexes, which was similar to other reports [15,16,25,27]. However, some studies reported better immunological response among female patients [22,28]. Whether HCV co-infection adversely affects HIV treatment outcomes remains controversial [16,29,30]. In this study, HCV seropositivity was not associated with higher risk of immunological failure. Due to the high adherence rate among patients evaluable for immunological failure (98.7% had adherence ≥ 95%), we could not demonstrate the association between poor adherence and immunological failure.

Our study has several limitations. First, HIV-1 RNA was not monitored at baseline in all patients so we couldn't evaluate the higher baseline HIV-1 RNA viral load as a predictor for virological failure. Second, the HIV-1 RNA monitoring was not regularly performed in all patients, therefore, we could not calculate the accurate time of treatment failure. It is also possible that some patients may already have virological failure at the time we evaluated the immunological criteria and thus misinterpret the true CD4 cell count response. Third, assessment and documentation of patient's adherence to cART was not standardized. However, this reflected the usual clinical practice and adherence was still the strongest predictor for virological failure. Fourth, this is a retrospective cohort study that the accuracy of analysis depends on the completeness of data entry into the database. Finally, this study is limited to a single cohort, the findings should be confirmed in other clinical settings.

In summary, this analysis of a cohort of HIV-infected patients under usual clinical care showed that predictors of immunological and virological failure can be identified using data routinely recorded. The risk factors of virological failure were poor adherence of cART and younger age, whereas those of immunological failure were low baseline CD4 count and slow CD4 cell recovery. This study demonstrated that even in resource-limited settings, the high rate of success could be expected in the cohort with good and sustainable drug adherence.

## Competing interests

The authors declare that they have no competing interests.

## Authors' contributions

NK participated in data collection, performed the statistical analysis, and drafted the manuscript. RC participated in the design of the study, performed the statistical analysis, and drafted the manuscript. KS and TS revised manuscript critically for important intellectual content. All authors read and approved the final manuscript.

## References

[B1] Ungass Country Progress Report Thailand Reporting Period January 2008-December 2009 http://www.unaids.org/en/dataanalysis/monitoringcountryprogress/2010progressreportssubmittedbycountries/thailand_2010_country_progress_report_en.pdf

[B2] Chasombat S Lertpiriyasuwat C Thanprasertsuk S Suebsaeng L Lo YR The National Access to Antiretroviral Program for PHA (NAPHA) in Thailand Southeast Asian J Trop Med Public Health 2006 37 704 15 17121296

[B3] Kiertiburanakul S Khongnorasat S Rattanasiri S Sungkanuparph S Efficacy of a generic fixed-dose combination of stavudine, lamivudine and nevirapine (GPO-VIR) in Thai HIV-infected patients J Med Assoc Thai 2007 90 237 43 17375626

[B4] Hogg RS Yip B Kully C Craib KJ O'Shaughnessy MV Schechter MT Montaner JS Improved survival among HIV-infected patients after initiation of triple-drug antiretroviral regimens CMAJ 1999 160 659 65 10102000 PMC1230111

[B5] Mocroft A Vella S Benfield TL Chiesi A Miller V Gargalianos P d'Arminio Monforte A Yust I Bruun JN Phillips AN Lundgren JD Changing patterns of mortality across Europe in patients infected with HIV-1. EuroSIDA Study Group Lancet 1998 352 1725 30 10.1016/S0140-6736(98)03201-2 9848347

[B6] Palella FJ Jr Delaney KM Moorman AC Loveless MO Fuhrer J Satten GA Aschman DJ Holmberg SD Declining morbidity and mortality among patients with advanced human immunodeficiency virus infection. HIV Outpatient Study Investigators N Engl J Med 1998 338 853 60 10.1056/NEJM199803263381301 9516219

[B7] Hogg RS Yip B Kully C Craib KJ O'Shaughnessy MV Schechter MT Montaner JS Improved survival among HIV-infected patients after initiation of triple-drug antiretroviral regimens CMAJ 1999 160 659 65 10102000 PMC1230111

[B8] Rajasekaran S Jeyaseelan L Vijila S Gomathi C Raja K Predictors of failure of first-line antiretroviral therapy in HIV-infected adults: Indian experience AIDS 2007 21 Suppl 4 S47 53 10.1097/01.aids.0000279706.24428.78 17620752

[B9] Robbins GK Daniels B Zheng H Chueh H Meigs JB Freedberg KA Predictors of antiretroviral treatment failure in an urban HIV clinic J Acquir Immune Defic Syndr 2007 44 30 7 10.1097/01.qai.0000248351.10383.b7 17106280 PMC2365745

[B10] Tuboi SH Harrison LH Sprinz E Albernaz RK Schechter M Predictors of virologic failure in HIV-1-infected patients starting highly active antiretroviral therapy in Porto Alegre, Brazil J Acquir Immune Defic Syndr 2005 40 324 8 10.1097/01.qai.0000182627.28595.01 16249707

[B11] Anekthananon T Ratanasuwan W Techasathit W Sonjai A Suwanagool S Safety and efficacy of a simplified fixed-dose combination of stavudine, lamivudine and nevirapine (GPO-VIR) for the treatment of advanced HIV-infected patients: a 24-week study J Med Assoc Thai 2004 87 760 7 15521230

[B12] Kozal MJ Hullsiek KH Macarthur RD Berg-Wolf M Peng G Xiang Y Baxter JD Uy J Telzak EE Novak RM The Incidence of HIV drug resistance and its impact on progression of HIV disease among antiretroviral-naive participants started on three different antiretroviral therapy strategies HIV Clin Trials 2007 8 357 70 10.1310/hct0806-357 18042501

[B13] Srasuebkul P Ungsedhapand C Ruxrungtham K Boyd MA Phanuphak P Cooper DA Law MG Predictive factors for immunological and virological endpoints in Thai patients receiving combination antiretroviral treatment HIV Med 2007 8 46 54 10.1111/j.1468-1293.2007.00427.x 17305932

[B14] Kabugo C Bahendeka S Mwebaze R Malamba S Katuntu D Downing R Mermin J Weidle PJ Long-term experience providing antiretroviral drugs in a fee-for-service HIV clinic in Uganda: evidence of extended virologic and CD4+ cell count responses J Acquir Immune Defic Syndr 2005 38 578 83 10.1097/01.qai.0000134742.26338.2f 15793369

[B15] Kaufmann GR Perrin L Pantaleo G Opravil M Furrer H Telenti A Hirschel B Ledergerber B Vernazza P Bernasconi E Rickenbach M Egger M Battegay M CD4 T-lymphocyte recovery in individuals with advanced HIV-1 infection receiving potent antiretroviral therapy for 4 years: the Swiss HIV Cohort Study Arch Intern Med 2003 163 2187 95 10.1001/archinte.163.18.2187 14557216

[B16] Moore RD Keruly JC CD4+ cell count 6 years after commencement of highly active antiretroviral therapy in persons with sustained virologic suppression Clin Infect Dis 2007 44 441 6 10.1086/510746 17205456

[B17] Parienti JJ Massari V Descamps D Vabret A Bouvet E Larouze B Verdon R Predictors of virologic failure and resistance in HIV-infected patients treated with nevirapine- or efavirenz-based antiretroviral therapy Clin Infect Dis 2004 38 1311 6 10.1086/383572 15127346

[B18] Ammassari A Trotta MP Murri R Castelli F Narciso P Noto P Vecchiet J D'Arminio Monforte A Wu AW Antinori A Correlates and predictors of adherence to highly active antiretroviral therapy: overview of published literature J Acquir Immune Defic Syndr 2002 31 Suppl 3 S123 7 12562034 10.1097/00126334-200212153-00007

[B19] McNabb J Ross JW Abriola K Turley C Nightingale CH Nicolau DP Adherence to highly active antiretroviral therapy predicts virologic outcome at an inner-city human immunodeficiency virus clinic Clin Infect Dis 2001 33 700 5 10.1086/322590 11486292

[B20] Perno CF Ceccherini-Silberstein F De Luca A Cozzi-Lepri A Gori C Cingolani A Bellocchi MC Trotta MP Piano P Forbici F Scasso A Vullo V d'Arminio Monforte A Antinori A Virologic correlates of adherence to antiretroviral medications and therapeutic failure J Acquir Immune Defic Syndr 2002 31 Suppl 3 S118 22 12562033 10.1097/00126334-200212153-00006

[B21] Turner BJ Adherence to antiretroviral therapy by human immunodeficiency virus-infected patients J Infect Dis 2002 185 Suppl 2 S143 51 12001036 10.1086/340197

[B22] Lucas GM Chaisson RE Moore RD Highly active antiretroviral therapy in a large urban clinic: risk factors for virologic failure and adverse drug reactions Ann Intern Med 1999 131 81 7 10419445 10.7326/0003-4819-131-2-199907200-00002

[B23] Glass TR De Geest S Weber R Vernazza PL Rickenbach M Furrer H Bernasconi E Cavassini M Hirschel B Battegay M Bucher HC Correlates of self-reported nonadherence to antiretroviral therapy in HIV-infected patients: the Swiss HIV Cohort Study J Acquir Immune Defic Syndr 2006 41 385 92 10.1097/01.qai.0000186371.95301.52 16540942

[B24] Panel on Antiretroviral Guidelines for Adults and Adolescents. Guidelines for the use of antiretroviral agents in HIV-1-infected adults and adolescents. Department of Health and Human Services 2009 1 161 http://www.aidsinfo.nih.gov/ContentFiles/AdultandAdolescentGL.pdf

[B25] Mezzaroma I Carlesimo M Pinter E Alario C Sacco G Muratori DS Bernardi ML Paganelli R Aiuti F Long-term evaluation of T-cell subsets and T-cell function after HAART in advanced stage HIV-1 disease AIDS 1999 13 1187 93 10.1097/00002030-199907090-00006 10416521

[B26] Aiuti F Mezzaroma I Failure to reconstitute CD4+ T-cells despite suppression of HIV replication under HAART AIDS Rev 2006 8 88 97 16848276

[B27] Lawn SD Myer L Bekker LG Wood R CD4 cell count recovery among HIV-infected patients with very advanced immunodeficiency commencing antiretroviral treatment in sub-Saharan Africa BMC Infect Dis 2006 6 59 10.1186/1471-2334-6-59 16551345 PMC1435908

[B28] Hunt PW Deeks SG Rodriguez B Valdez H Shade SB Abrams DI Kitahata MM Krone M Neilands TB Brand RJ Lederman MM Martin JN Continued CD4 cell count increases in HIV-infected adults experiencing 4 years of viral suppression on antiretroviral therapy AIDS 2003 17 1907 15 10.1097/00002030-200309050-00009 12960823

[B29] Braitstein P Zala C Yip B Brinkhof MW Moore D Hogg RS Montaner JS Immunologic response to antiretroviral therapy in hepatitis C virus-coinfected adults in a population-based HIV/AIDS treatment program J Infect Dis 2006 193 259 68 10.1086/498908 16362890

[B30] Law WP Duncombe CJ Mahanontharit A Boyd MA Ruxrungtham K Lange JM Phanuphak P Cooper DA Dore GJ Impact of viral hepatitis co-infection on response to antiretroviral therapy and HIV disease progression in the HIV-NAT cohort AIDS 2004 18 1169 77 10.1097/00002030-200405210-00010 15166532

